# Research on Thermal Stability and Flammability of Wood Scob-Based Loose-Fill Thermal Insulation Impregnated with Multicomponent Suspensions

**DOI:** 10.3390/ma17122809

**Published:** 2024-06-08

**Authors:** Nerijus Augaitis, Saulius Vaitkus, Agnė Kairytė, Sigitas Vėjelis, Jurga Šeputytė-Jucikė, Giedrius Balčiūnas, Arūnas Kremensas

**Affiliations:** Laboratory of Thermal Insulating Materials and Acoustics, Institute of Building Materials, Faculty of Civil Engineering, Vilnius Gediminas Technical University, Linkmenų St. 28, 08217 Vilnius, Lithuania; saulius.vaitkus@vilniustech.lt (S.V.); agne.kairyte@vilniustech.lt (A.K.); sigitas.vejelis@vilniustech.lt (S.V.); jurga.seputyte-jucike@vilniustech.lt (J.Š.-J.); giedrius.balciunas@vilniustech.lt (G.B.); arunas.kremensas@vilniustech.lt (A.K.)

**Keywords:** loose-fill thermal insulation, liquid glass, expandable graphite, tung oil, coating, wood composite, fire resistance, smoldering

## Abstract

Loose-fill thermal composite insulation produced from surface-modified wood scobs has been explored as a potential fire-resistant material for building envelopes. This work involves fire resistance behavior comparisons between four coating systems consisting of liquid glass, liquid glass-tung oil, liquid glass-expandable graphite, and liquid glass-tung oil-expandable graphite. The techniques of thermogravimetric and differential thermogravimetric analyses, gross heat combustion via a calorimetric bomb, cone calorimetry, SEM imaging of char residues, and energy dispersive spectrometry for elemental analysis, as well as propensity to undergo continuous smoldering, were implemented. The coating technique resulted in greater thermal stability at a higher temperature range (500–650 °C) of the resulting loose-fill thermal composite insulation, reduced flame-damaged area heights after the exposure of samples at 45° for 15 s and 30 s, with a maximum of 49% decreased gross heat combustion, reduced heat release and total smoke release rates, improved char residue layer formation during combustion and changed smoldering behavior due to the formation of homogeneous and dense carbon layers. The results showed that the highest positive impact was obtained using the liquid glass and liquid glass-expandable graphite system because of the ability of the liquid glass to cover the wood scob particle surface and form a stable and strong expanding carbon layer.

## 1. Introduction

Construction and building industries, as well as their activities, are the main contributors to global carbon dioxide emissions through operational and embodied energy use [1]. It consumes a large amount of non-renewable resources, and many products are composed of materials that are harmful to human health and contaminate the indoor environment [2]. The insulation of the building envelopes is one of the most effective measures to reduce operational energy use, which ensures the benefits of energy use for cooling and heating. Conventional thermal insulation materials existing in the market are generally petroleum-based materials, e.g., expanded polystyrene, extruded polystyrene, polyurethane, polyisocyanurate, and phenolic foams. These materials have low thermal conductivity values, which assures high thermal insulation performance [3], low density, and affordability. However, the environmental impact of their production processes is high. Consequently, the application of building insulation from natural renewable resources with minimal production processing is very important in the development of a more sustainable and healthier environment [4].

Lately, many studies have been conducted on the application and development of bio-based or natural fiber insulation materials as a replacement for petroleum-based materials, such as cotton [5], cork [6], flax and hemp [7], wood fiber [8], coconut [9], rice straw [10], cellulose [11], sheep wool [12], etc. The main advantage of plant-based materials is that they have the ability to capture atmospheric carbon dioxide through photosynthesis. Therefore, the use of such materials in the construction industry can reduce the net embodied carbon dioxide emissions of the whole building [13]. With a lower or potentially negative carbon footprint as well as no or fewer health issues during application, these materials can deliver high performance that could be comparable to other insulation materials.

In 2024, the Ministry of the Environment of Lithuania established a requirement for public buildings to be constructed from wood and organic plant-based materials, which meant 50% of the construction materials of the entire structure would be composed of these materials. However, it should be noted that when using wood or organic plant-based materials, special attention to the fire safety of such buildings is necessary. When designing and constructing buildings, the flammability classification of such building materials [14] becomes very important. In order for the materials to be thermally stable and meet the flammability requirements, flame retardants should be used.

Currently, the development of bio-based flame retardants has been widely implemented due to their low cost, renewability, and environmental friendliness. Many natural bio-based materials, such as vanillin [15], adenosine [16], phytic acid [17,18], vegetable oils [19], and lignin [20], can be used as raw materials for flame retardant bases applied on the surfaces of wood and wood-based composites. Conventional film coatings that form the base materials for wood surfaces include epoxy resins [21], amino resins [22], polyurethane resins [23], and acrylic resins [24]. However, they are not considered environmentally friendly. Therefore, this aspect should be taken into consideration when seeking an alternative base for a bio-based flame-retardant system.

One of the flame retardants is expandable graphite (EG). It is widely used as a green, eco-friendly flame retardant, mainly in flame-retardant polymers such as propylene [25], polystyrene [26], polyamide [27], and polyurethane [28]. Moreover, EG was also implemented as a flame retardant in wood–plastic composites [29] as EG forms “worm-like” char layers at high temperatures, which cover the surface of the material to suppress heat and mass transfer [30,31]. However, pure EG as a flame retardant has some drawbacks. Firstly, it has poor interfacial adhesion between analyzed surfaces, thus deteriorating the mechanical and water resistance performance of composite materials [32]. Secondly, the “popcorn” and “effect wick” of EG, when used alone, could also reduce the flame-retardant effect [33]. Finally, the flame-retardant efficacy can only be achieved with the addition of a higher amount of EG.

In order to overcome these drawbacks, the application of wood scobs (WSs) as a loose-fill thermal insulation material and its surface modification with sodium silicate liquid glass (LG) and tung oil (TO) to achieve a proper interface with EG was proposed in a previous work by Augaitis et al. [34]. The results showed a superior improvement in thermal conductivity and water resistance. However, there is a lack of knowledge on the thermal stability and fire resistance properties of the obtained and similar loose-fill thermal insulation composites. Therefore, the aim of the current study was to determine the synergistic impact of EG, LG, and TO on the fire resistance properties of WS-based loose-fill thermal insulation composites.

## 2. Materials and Methods

### 2.1. Raw Materials and Composition of the Composites

LG was supplied from Lerochem, Klaipėda, Lithuania. It was composed of 9.3% Na_2_O and 28.0% SiO_2_; its density at 20 °C was 1.38 g/cm^3^, and the molar ratio was 3.1. TO was purchased from Pro Colore, Vilkaviškis, Lithuania. EG ES 350 F5 was supplied from Qingdao Kropfmuehl Graphite Co., Ltd., Qingdao, China. It had the following characteristics: a carbon content of 98%, a rate of expansion of 350–700 cm^3^/g, and a temperature of expansion of 180–240 °C. Pinewood WS was obtained from a forest in the Dzūkija region, Alytus, Lithuania. The main characteristics of WS were as follows: the average bulk density was 83.0 ± 6.8 kg/m^3^, and the moisture content was 12.1 ± 1.9%. The granulometry of the WS particles can be found in a study by Augaitis et al. [34].

The results of thermal conductivity, short-term water absorption, contact angle, and water vapor permeability in [34] showed that the appropriate multicomponent suspension for the WS coating was LG/TO/EG. However, for the proper and more precise interpretation of the thermal stability and fire resistance results, interim compositions of loose-fill thermal insulation composites WS, WS/LG, WS/LG/TO, and WS/LG/EG were selected, and their compositions are presented in Table 1.

Multicomponent suspensions from LG, LG/TO, LG/EG, and LG/TO/EG were blended with a high-speed mixer for 1 min at 16,000 rpm. The obtained multicomponent suspensions were then mixed with WS particles until homogeneous coverage was achieved. The LG, LG/TO, LG/EG, and LG/TO/EG-coated WS particles were vacuumed at 1 bar for 10 min and left to dry at 23 ± 5 °C and 50 ± 5% relative air humidity for 1 week.

### 2.2. Methods

#### 2.2.1. Testing Methods

Thermogravimetric analysis (TGA) and differential thermogravimetric analysis (DTG) were performed using the TG 209 F1 analyzer (Netzsch Group, Selb, Germany). The temperature was measured with a K-type thermocouple. The analysis was carried out in the temperature range from 25 °C to 900 °C. The temperature rising speed was 10 °C/min. The analysis was carried out in an air atmosphere for one sample of each composition.

In order to determine the ignitability of the loose-fill thermal insulation composites, the specimens were subjected to the direct impingement of a flame in accordance with the requirements of ISO 11925-2:2020 [35] using the KBK 917 apparatus (Netzsch Group, Selb, Germany). Six vertical specimens for each composition with dimensions of 200 mm × 100 mm × 50 mm were directly exposed to a flame at an angle of 45°. The flame exposure durations during the test were 15 s and 30 s. The test was carried out in an environment of 23 ± 5 °C and 50 ± 5% relative air humidity.

The gross heat of combustion was measured using a KL-12 Mn calorimeter (Precyzja Group, Bydgoszcz, Poland). During the test, the calorific values of different suspension-coated loose-fill thermal insulation composites were determined by performing three tests from one type of suspension-coated loose-fill thermal insulation composite, thus determining the calorific values and calculating them as the arithmetic mean of the three test values in accordance with the requirements of the EN ISO 1716:2018 standard [36]. The parameters used for the tests were as follows: 99.5% oxygen purity and 3.0–3.5 MPa pressure.

The heat release rate (HHR), total smoke production per unit area (TSR), carbon monoxide yield (COY), and carbon dioxide yield (CO_2_Y) were determined according to ISO 5660-1:2015 [37] with a cone calorimeter Cone 2a (Atlas Electric Devices Co., Chicago, IL, USA). The parameters used for the test were a heat flow of 35 kW/m^2^, a surface area of specimens of 90 cm^2^, and an overall testing time of 1200 s. The test was carried out for three samples of each composition.

The propensity to undergo continuous smoldering was determined based on the requirements of EN 16733:2016 [38] with a TSP-16733 apparatus (Taurus Instruments, Fridingen, Germany). WS particles coated with a multicomponent suspension were placed in a vertical metal test frame made of a metal-galvanized mesh having a size of 800 mm × 300 mm × 100 mm, and the eyelet size was 9 ± 1 mm. Then, 0.85 L/min 95% propane gas was used to ensure combustion. Next, 6 K-type thermocouples were used to measure the temperature. The thermocouples were placed vertically at 100 mm intervals through the middle of the test frame, inserting the thermocouples 50 mm into the specimen. The surface was exposed to a flame for 15 min. The test was considered successful if the readings of all six thermocouples did not increase for 60 min and if the readings of each thermocouple did not exceed 50 °C. In total, two samples of each composition were tested.

For the analysis of the char residues after burning, a scanning electron microscope (Helios NanoLab 650 (Oxford Instruments, Abingdon, UK)) was used. Before imaging, the samples were coated with a thin gold layer. The elemental composition of the char residues was determined using an EDS system (INCAEnergy (Oxford Instruments, Abingdon, UK)) and an X-max detector. One sample of each composition was tested in order to determine the elemental composition.

#### 2.2.2. Statistical Methods

An analysis of variance (ANOVA-Stat.Soft, v.8) was used to analyze the experimental data. It was used to compare the averages of the studied variables. The ANOVA made it possible to assess whether the averages of different groups were the same. A one-factor variance analysis was used, using the F distribution and choosing a significance level of *p* = 0.05. Additionally, the coefficient of determination, R^2^, and adjusted coefficient of determination, R^2^, were used to represent the part of the variance of the dependent variable explained by the factors.

## 3. Results and Discussion

**Figure 1:**
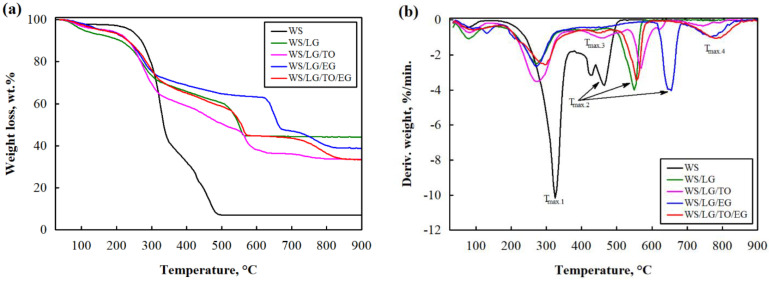
Thermal stability of loose-fill thermal insulation material composites: (**a**) thermogravimetric analysis; (**b**) differential thermogravimetric analysis.

Figure 1 presents the curves obtained from the thermogravimetric (Figure 1a) and differential thermogravimetric (Figure 1b) analyses, while the results from the main endothermic peak temperatures, as well as the amount of char residues at 900 °C are presented in Table 2. The WS coatings were characterized using the thermal degradation intensities characteristic of wood. At temperatures up to 105 °C, the moisture contained in the samples was removed, and the sample lost 2.3% of its mass.

Meanwhile, in the temperature range from 220 °C to 350 °C, the thermal depolymerization of hemicellulose occurred with the largest mass loss, i.e., 54.5% (T_max.1_ = 325 °C), and from 350 °C to 500 °C, the decomposition of the cellulose glycosidic bond, pectin, and lignin appeared with a 48.5% mass loss (T_max.2_ = 465 °C). After coating the WS particles with LG (all compositions), a shift of T_5%_ and T_max.1_ to the lower temperature was observed. The authors Khoathane et al. [39] observed that the alkaline treatment of plant-based fillers and fibers removed part of the hemicellulose and pectins, so a high amount of LG in the suspensions reduced the thermal stability of all the loose-fill thermal insulation material composites at temperatures up to 350 °C. EG was also expected to increase the thermal stability of multicomponent suspensions at lower temperatures, but according to some authors [40,41], EG activates the decomposition of composites due to the sulfuric acid present in EG layers, but in these temperatures, the rate of decomposition of all the samples decreased almost three times due to the effect of temperature on EG oxidation, which occurs due to the reduction reaction with H_2_SO_4_ (Equation (1)), during which CO_2_, H_2_O, and SO_2_ gases are released, reducing the negative effect of temperature [42].

(1) $$C+2H_{2}{SO}_{4}\to {CO}_{2}↑+{2H}_{2}O↑+{2SO}_{2}↑$$

In multicomponent suspensions with TO (WS/LG/TO and WS/LG/TO/EG) in the temperature range of 300–490 °C, triglyceride decomposition was observed at the intensity marked T_max.3_. From the results presented in Table 2, it can be observed that the intensity of triglyceride decomposition shifted towards higher temperatures, i.e., from 465 °C to 480 °C with the additional use of EG (the WS/LG/TO/EG composition).

In the WS sample, the two intensities observed in the temperature range of 350–500 °C, marked as one T_max.2_, turned into one due to the effect of LG, which is basically related to the probable removal of pectins and overlap of the cellulose and lignin intensities. It is interesting that compared to the WS sample, the WS/LG and WS/LG/TO sample thermal decomposition temperature T_max.2_ intensities increased by 85 °C and 105 °C, respectively. This may have been because LG swells at temperatures above 500 °C and acts as a barrier to temperature effects.

It can also be observed that when EG is used in a multicomponent suspension (the WS/LG/EG composition), it has a very effective impact. Compared to WS, the thermal stability of such samples at higher temperatures increased by as much as 190 °C. Although EG did not show a positive effect at lower temperatures, at higher temperatures, there was a significant shift of T_max.2_ towards higher temperatures was observed in the WS/LG/EG and WS/LG/TO/EG loose-fill thermal composite insulation materials. The synergy between LG and EG was clearly observed and manifested by the ability of these two components to swell under the influence of temperature and form a double LG/EG barrier at high temperatures.

When further analyzing the results presented in Table 2, it can be observed that WS had the lowest amount of char residues after exposure at 900 °C, but when WS was coated with the LG, LG/TO, LG/EG, and LG/TO/EG suspensions, these amounts increased by 37.0%, 26.5%, 31.9%, and 26.1%, respectively. It means that WS, with various types of coatings, generates a larger amount of char residues, which reduce pyrolysis reactions and inhibit the formation of flammable gases.

The observed intensity, T_max.4_, in the temperature range of 650–820 °C in the loose-fill thermal composite insulation materials with the compositions of WS/LG/EG and WS/LG/TO/EG indicates the presence of strongly bonded oxygen-containing functional groups in EG, which were not removed during EG expansion at lower temperatures [43].

**Figure 2:**
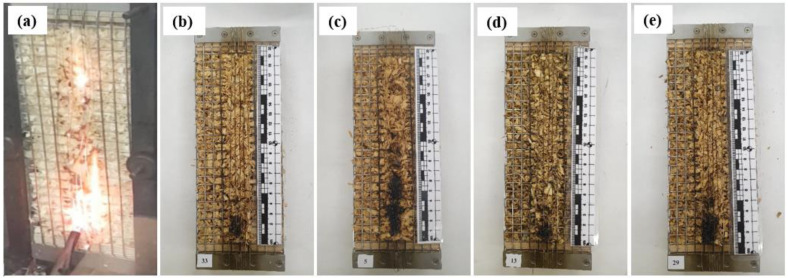
Samples of loose-fill thermal composite insulation materials after 15 s of flame impact: (**a**) WS; (**b**) WS/LG; (**c**) WS/LG/TO; (**d**) WS/LG/EG; and (**e**) WS/LG/TO/EG.

**Figure 3:**
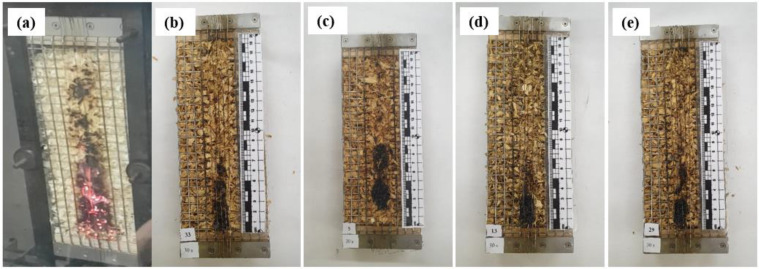
Samples of loose-fill thermal composite insulation materials after 30 s of flame impact: (**a**) WS; (**b**) WS/LG; (**c**) WS/LG/TO; (**d**) WS/LG/EG; and (**e**) WS/LG/TO/EG.

In order to determine the behavior of the developed loose-fill thermal composite insulation materials under direct flame exposure, flammability tests were performed. Figure 2 and Figure 3 show the samples exposed to an open flame for 15 s and 30 s.

According to the research results, it was observed that the WS sample continued to burn after 15 s (Figure 2a) and 30 s (Figure 3a) after removing the active flame source, and the surface area damaged by the flame was large. It was also observed that the sample smoldered after the flame was extinguished. All loose-fill thermal composite insulation materials, regardless of the composition of the coating used to cover the WS particles, had a completely different flaming behavior than that of the uncoated WS particles. Figure 2b–e and Figure 3b–e show that when the flame source was removed after 15 s and 30 s, all loose-fill thermal composite insulation materials did not support combustion or exhibit self-extinguishment, and the flame-damaged surface area was reduced to a minimum. Interestingly, the flame performance of the loose-fill thermal composite insulation coating with TO was similar to that of the rest of the coatings, which suggests that LG has the greatest positive effect on multicomponent coatings.

**Figure 4:**
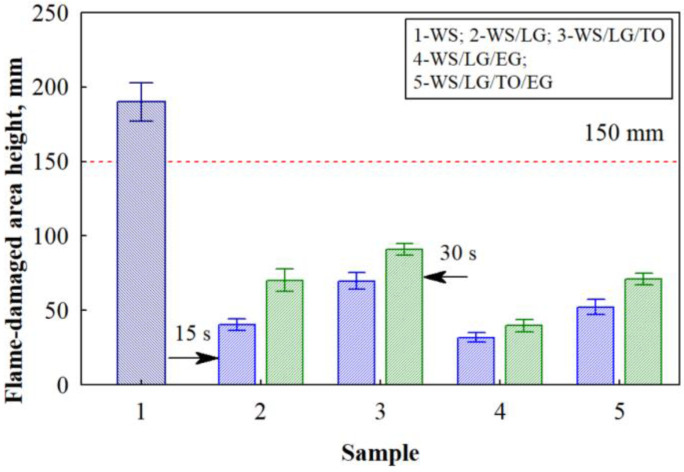
Flame-damaged area height after 15 s and 30 s of burning the loose-fill composite thermal insulation materials.

In Figure 4, the average values of the flame heights when the samples were exposed to an open flame are provided. It was observed that the height of the flame-damaged area of the WS samples reached the limit of ~190 ± 12.1 mm. It was also observed that the WS/LG loose-fill thermal composite insulation material exhibited flame heights of 40.3 ± 3.67 mm and 70.3 ± 7.20 mm after removing the flame source after 15 s and 30 s, respectively. Compared with WS, the flame heights of the WS/LG loose-fill thermal composite insulation material decreased by 79% and 63% after 15 s and 30 s, respectively, indicating the effectiveness of the LG coating and its ability to protect the coated plant-based materials from fire.

Meanwhile, the WS/LG/TO loose-fill thermal composite insulation material reached the highest flame-damaged area heights. After removing the flame source after 15 s and 30 s, the flame heights were 69.8 ± 5.42 mm and 90.8 ± 3.66 mm, respectively. Such results for this loose-fill thermal composite insulation material are not surprising because TO oxidizes rapidly when exposed to high temperatures, thus promoting the combustion process in the material coated with it. It is also established that all loose-fill thermal composite insulation materials, regardless of their coating, extinguish, do not smoke, and the flame does not reach the standardized limit of 150 mm. The research shows that the WS/LG/EG samples had the lowest flame heights, i.e., 31.3 ± 2.99 mm and 39.7 ± 4.08 mm after 15 s and 30 s, respectively. As shown by the thermogravimetric and differential thermogravimetric analyses, both LG and EG play the role of a barrier to high-temperature changes. A similar effect of these two components can also be observed during the tests of the loose-fill composite thermal insulation material coated with the LG/TO/EG multicomponent suspension. After removing the flame source after 15 s and 30 s, the flame heights were 52.3 ± 4.68 mm and 71.0 ± 3.85 mm, respectively.

The statistical analysis of the results obtained after 15 s, excluding the uncoated WS, showed that the average values of the flame heights differed. The F criterion was 88.2, with *p* > 0. This indicates a statistically significant difference in the results of the subjects. Accordingly, the coefficient of determination was R^2^ = 0.930, and the corrected coefficient of determination was R^2^ = 0.940. After 30 s, the average values of the damaged area heights were also different. The F criterion was 111, with *p* > 0. This also indicates a statistically significant difference in the results of the subjects. Accordingly, the coefficient of determination was R^2^ = 0.943, and the corrected coefficient of determination was R^2^ = 0.935.

**Figure 5:**
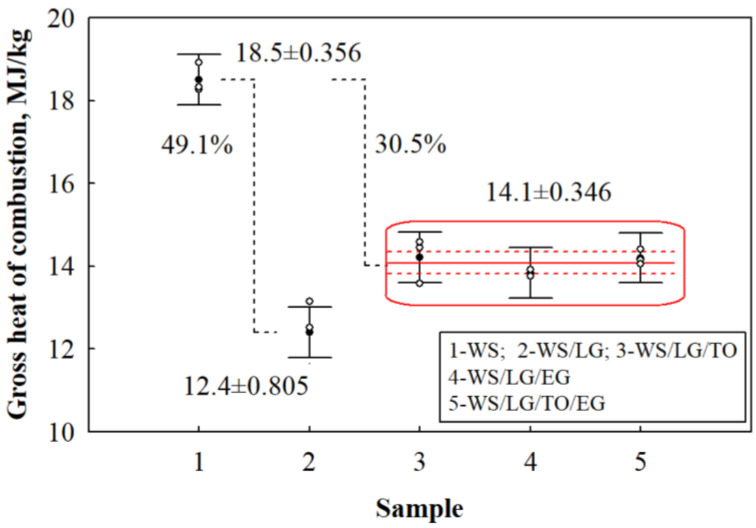
Results of the gross heat of combustion of loose-fill thermal composite insulating materials.

After the additional testing of the loose-fill thermal composite insulation materials with a calorimetric bomb, it was observed that using multicomponent suspensions for coating, it is possible to reduce the gross heat of combustion of plant-based materials (Figure 5). This method determines how much heat is released from a completely burned material. The less heat the burning material emits, the greater the efficiency potential of the multicomponent suspension in reducing the amount of heat emitted during combustion and the slower the flame spread.

The obtained research results show that the gross heat of combustion of WS was equal to 18.5 MJ/kg. Accordingly, the gross heat of combustion of the WS/LG loose-fill thermal composite insulation material reached 12.4 MJ/kg. It can be seen that compared to WS, the value of the gross heat of combustion of the WS/LG loose-fill thermal composite insulation material decreased by 49.1%. The difference in the averaged values between the WS and WS/LG/TO, WS/LG/EG, and WS/LG/TO/EG samples was 30.5%, and the performed mathematical analysis showed that the WS/LG/TO, WS/LG/EG, and WS/LG/TO/EG loose-fill thermal composite insulation materials did not significantly differ and their gross heat of combustion was equal to 14.1 MJ/kg. Therefore, it can be assumed that LG had the highest contribution to the gross heat of combustion due to its ability to cover the particles’ surface and form a stable and strong expanding carbon layer.

In order to evaluate whether the gross heat of combustion of WS differed after coating it with different multicomponent suspension coatings (samples 1–5, Figure 5), a variance analysis was performed, the purpose of which was to check whether the difference between the average values of the obtained results was significant. The analysis showed that the difference in the average values of the gross heat of combustion of samples 1–5 was significant. The F criterion was 70.4, with *p* = 0, the coefficient of determination was R^2^ = 0.965, and the corrected coefficient of determination was $\bar{R}$^2^ = 0.952. Repeated analyses showed no difference between samples 3–5 (F = 1.23, p = 0.357, R_2_ = 0.291 and $\bar{R}$^2^ = 0.0541).

**Figure 6:**
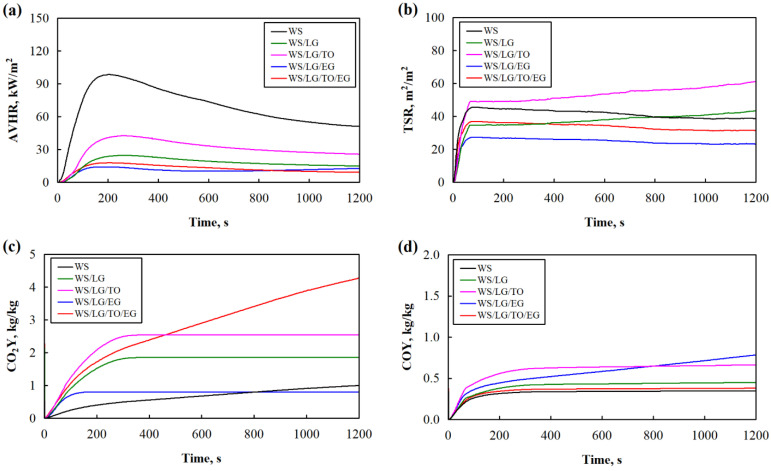
Cone calorimeter parameters of loose-fill thermal composite insulation materials: (**a**) heat release rate; (**b**) total smoke release; (**c**) CO_2_ yield; (**d**) CO yield.

One of the best ways to determine the behavior of products under the impact of a flame and obtain the corresponding characteristics during combustion is cone calorimeter testing; therefore, for the additional evaluation of the flammability characteristics of the loose-fill thermal composite insulation materials, the average heat release rate (AVHR), total smoke release (TSR), carbon dioxide yield (CO_2_Y), and carbon monoxide yield (COY) were determined, and the results of the change in these characteristics during combustion are presented in Figure 6, with the highest values achieved in Table 3.

Figure 6a shows that compared to WS, the AVHR curves of all the loose-fill thermal composite insulation materials were less intense, and the amount of heat released was lower throughout the test.

**Figure 7:**
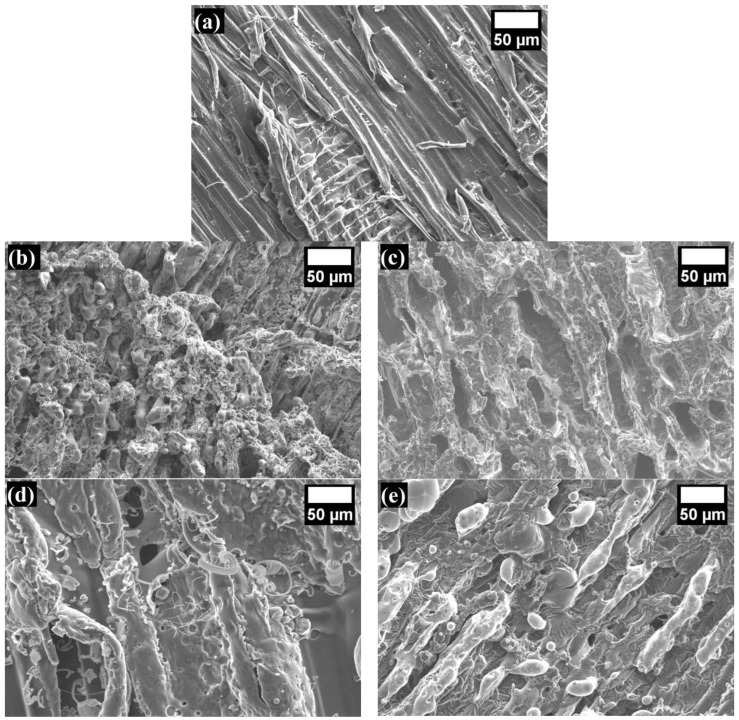
Microstructure images of the char residues of the burnt samples after the cone calorimeter test (magnification ×500): (**a**) WS; (**b**) WS/LG; (**c**) WS/LG/TO; (**d**) WS/LG/EG; and (**e**) WS/LG/TO/EG.

A protective carbon layer did not form on the surface of the WS particles during combustion (Figure 7a), so the highest pHRR value was observed (Table 3), but after coating the particles with the LG suspension, this parameter decreased by 75%. From Figure 7b, it can be observed that an uneven LG layer was formed on the surface of the WS particles during combustion, but it expanded in some places, which allowed the inhibition of the decomposition of the WS particles due to the phase transformation and crystallization of the Na–O–Si gel.

Therefore, the EDS test (Table 4) shows that all carbonaceous layers of the loose-fill thermal composite insulation materials contained Na and Si elements after combustion. The phase transformation of the amorphous glasses of various compositions for the formation of metastable structures under the influence of high temperature is discussed quite extensively in the works of [44,45], who found that an LG suspension is an excellent immobilizing medium for inorganic particles, which can contribute to the fire resistance of wood.

**Figure 8:**
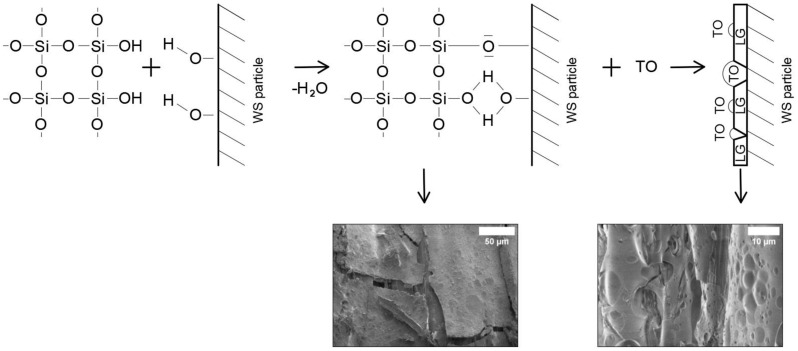
Proposed graphical interpretation of LG and TO coating formations on the surface of the WS particles.

It can be seen that the LG/TO coating had the least effect on the loose-fill thermal composite insulation materials, with a 57% reduction in the pHRR. Compared to the rest of the loose-fill thermal composite insulation materials, the changes in this parameter were caused by the fact that TO formed bubble-like films on the surface of the LG coating (Figure 8), which were not resistant to high temperatures.

It can be seen from Figure 7c that the loose-fill thermal composite insulation materials coated with the LG/TO multicomponent suspension were characterized by a porous carbon layer with cavities of various sizes. Such changes in the structure indicate that a less dense or not fully suitable carbon layer was formed during combustion, the lack of which led to burnt TO films in the multicomponent suspension coating. It can also be observed that the interaction of LG and EG during the formation of a coating on the surface of the WS particles led to a synergistic effect of heat release during the combustion of the materials, i.e., the pHRR value of the WS/LG/EG loose-fill thermal composite insulation material decreased by 85%, while for WS/LG it decreased by 75%. Due to the expansion of LG at high temperatures, a dense protective carbon layer was formed, the efficiency of which increased the ability of EG to change the volume and block the penetration of the flame into the deeper layers of the loose-fill thermal composite insulation material (Figure 7d). Interestingly, compared to WS, the loose-fill thermal composite insulation material coated with the multicomponent suspension LG/TO/EG exhibited a pHRR value that decreased by 82%. It was expected that the introduction of the TO component into the multicomponent suspension coating would have had a significant negative effect on the flammability characteristics, but as shown in Figure 7e, the carbon layer formed after the combustion of such loose-fill thermal composite insulation material was quite dense, and an expanded LG structure was observed. In the case of the LG/TO coating, no visible voids were formed, suggesting that EG protected TO from the adverse effects of the flame by changing its volume at high temperatures.

In order to ensure people’s safety in the event of a fire, it is extremely important to assess the amount of smoke and toxic substances released during the burning of building materials. Figure 6b presents the behavior of loose-fill thermal composite insulation materials with respect to total smoke release. Figure 6c illustrates the CO_2_ emissions, Figure 6d illustrates the CO emissions, and Table 3 shows the maximum values of the TSR, CO_2_, and CO obtained during combustion.

It can be seen that both the LG and LG/TO coatings increased the TSR by 1.1 and 1.6 times, respectively. This indicates that the LG coating had the greatest effect on smoke generation. A completely opposite trend in the results can be observed for the loose-fill thermal composite insulation materials with coatings containing EG (LG/EG and LG/TO/EG). It was observed that compared to WS particles, the TSR values of the WS/LG/EG and WS/LG/TO/EG loose-fill thermal composite insulation materials decreased from 36.6 m^2^/m^2^ to 23.3 m^2^/m^2^ and 31.4 m^2^/m^2^, respectively. Such a decrease in the average TSR values due to multicomponent suspensions with EG coatings can be explained by the physical formation of an extremely expanded and thermally stable carbonaceous layer (Figure 7d,e). Such a layer effectively stops the spread of fire and the formation of smoke, hindering the penetration and release of heat to and from the lower layers of the burning material, the movement of oxygen to the surface, and the transformation of low molecular mass decomposition products into gases [46]. It can be seen from Table 3 that the one-component coating (with only LG) and all the two-component coatings (LG/TO and LG/EG) resulted in increased CO emissions. For instance, compared to the WS particles, the parameter increased by 29%, 89%, and 126% in the presence of the LG, LG/TO, and LG/EG coatings, respectively. Meanwhile, the WS/LG/TO/EG loose-fill thermal composite insulation material showed similar CO emissions to the WS particles due to the previously mentioned ability of this coating to form a dense carbon layer that prevents the penetration of toxic gases from the material into the environment during combustion. When analyzing the CO_2_ emission results, it can be noted that the loose-fill thermal composite insulation materials with TO in the coating had the highest average values. Compared to the WS particles, the LG/TO and LG/TO/EG coatings increased the CO_2_ emissions by two and four times, respectively, while the CO emissions of loose-fill thermal composite insulation materials coated with the LG and LG/EG coatings changed only slightly. The increase in these values is attributed to both the TO thermal instability and CO_2_ release during EG expansion.

Additionally, statistical analyses were involved in order to evaluate the significance of the obtained results during cone calorimeter testing. It is shown in Table 3 that the average pHRR values of both the uncoated and LG, LG/EG, LG/TO, and LG/TO/EG-coated WS particles differed. Meanwhile, repeated analyses showed that the F criterion was 5.25, with *p* < 0.0837 for WS/LG/EG and WS/LG/TO/EG. This indicates a statistically insignificant difference in the results of the subject. Accordingly, the coefficient of determination was R^2^ = 0.567, and the corrected coefficient of determination was R^2^ = 0.459 for both loose-fill thermal composite insulation materials. Further, the average TSR values of both the uncoated and LG, LG/TO, LG/EG, and LG/TO/EG-coated WS particles differed. Repeated analyses showed that the F criterion was 1.74, with *p* < 0.257 for the WS and WS/LG loose-fill thermal composite insulation materials. This indicates a statistically insignificant difference in the results of the subject. Accordingly, the coefficient of determination was R^2^ = 0.303, and the corrected coefficient of determination was R^2^ = 0.128. Then, the average COY values of both the uncoated and LG, LG/TO, LG/EG, and LG/TO/EG-coated WS particles differed. Repeated analyses showed that the F criterion was 2.33, with *p* < 0.178 for the WS, WS/LG, and WS/LG/TO/EG loose-fill thermal composite insulation materials. This indicates a statistically insignificant difference in the results of the subject. Accordingly, the coefficient of determination was R^2^ = 0.437, and the corrected coefficient of determination was R^2^ = 0.249. Lastly, the average CO_2_Y values of both the uncoated and LG, LG/TO/LG/EG, and LG/TO/EG-coated WS particles differed. Repeated analyses showed that the F criterion of WS and WS/LG/EG was 2.62, with *p* < 0.181. This indicates a statistically insignificant difference in the results of the subject. Accordingly, the coefficient of determination was R^2^ = 0.396, and the corrected coefficient of determination was R^2^ = 0.245.

**Figure 9:**
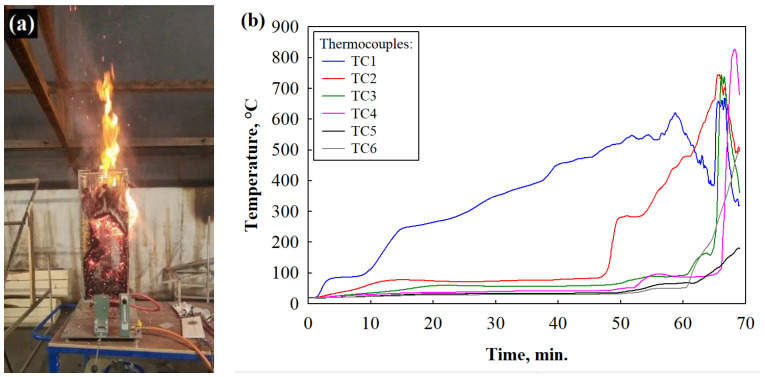

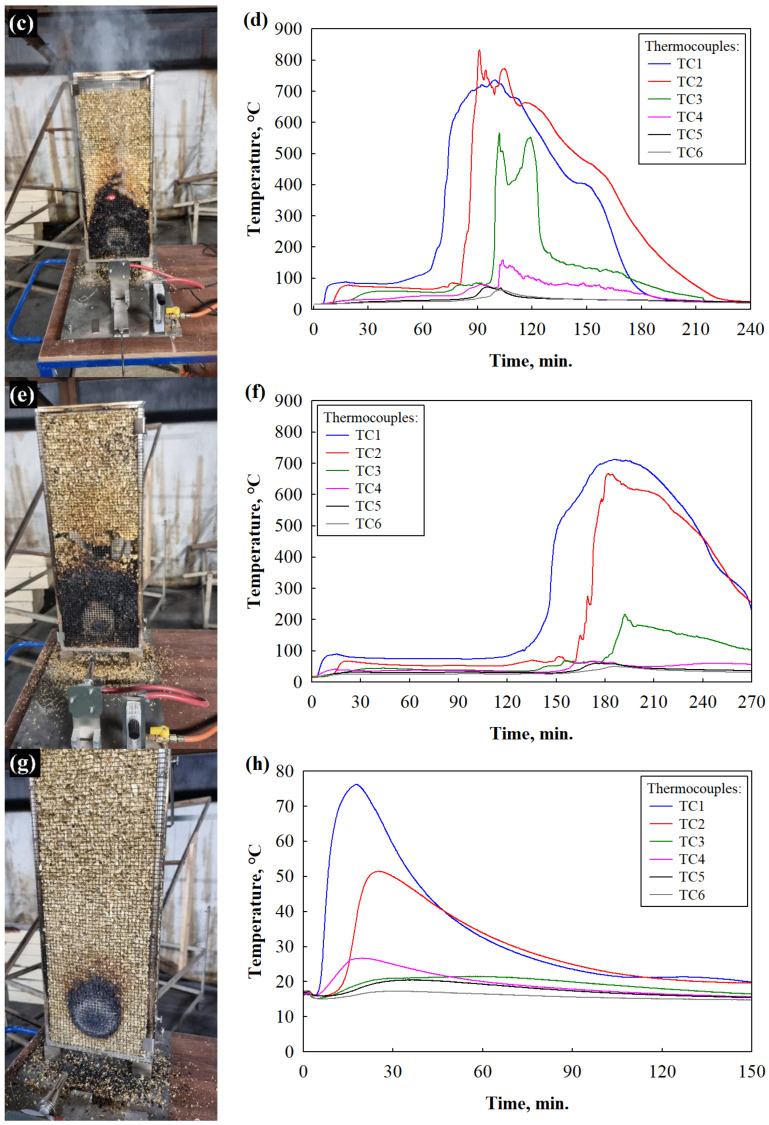

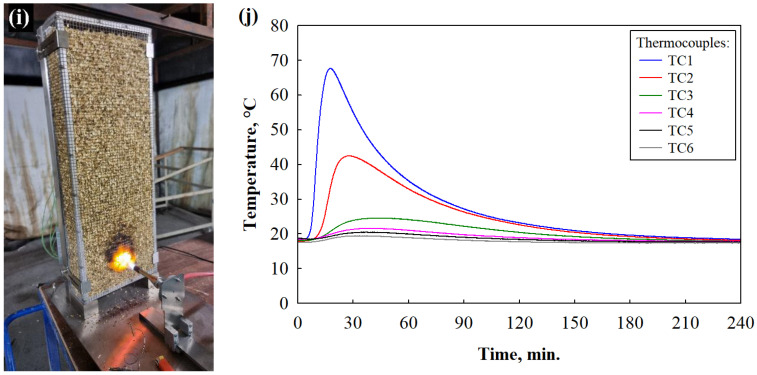
Smoldering test results of loose-fill thermal composite insulation materials: (**a**) WS sample during burning; (**b**) thermocouples’ results for the WS sample; (**c**) WS/LG sample during burning; (**d**) thermocouples’ results for the WS/LG sample; (**e**) WS/LG/TO sample during burning; (**f**) thermocouples’ results for the WS/LG/TO sample; (**g**) WS/LG/EG sample during burning; (**h**) thermocouples’ results for the WS/LG/EG sample; (**i**) WS/LG/TO/EG sample during burning; and (**j**) thermocouples’ results for the WS/LG/TO/EG sample.

Smoldering is one of the most dangerous properties of building materials. This process involves different simultaneous phenomena involving various chemical reactions interacting with the transfer of heat, mass, and inertia in the gas and solid phases. The rate of combustion is determined by various factors, such as the availability of oxygen, heat transfer, the nature of the material, and its chemical composition, so it is very important to evaluate the kinetics of the change in this parameter in materials that can have a direct impact on human health and life during a fire. The effectiveness of the selected loose-fill thermal composite insulation material coatings was evaluated after 60 min when the temperature of the material during combustion could not reach 50 °C. Figure 9 presents the combustion results of all the analyzed loose-fill thermal composite insulation materials. During the tests (Figure 9a,b), it became clear that the WS particles exposed to the flame burned uncontrollably and, after 60 min, reached a temperature of 564 °C and, when extinguished, smoked for a long time. Liang et al. [47] showed similar results of burning wood, therefore, in order to ensure the safe use of such materials in the construction industry, it is necessary to use coating or impregnation systems.

Figure 9c,d shows that the LG coating reduced the temperature of the loose-fill thermal composite insulation material up to 113 °C after 60 min but did not stop the smoldering process. The temperature rise in the lower layers (thermocouple TC1) was observed at about the 70th minute. A similar smoldering trend was observed in the LG/TO-coated loose-fill thermal composite insulation material (Figure 9e,f) when the temperature was reduced to 75 °C after 60 min, but a rise in temperature was observed after 130 min. It means that the one-component LG and two-component LG/TO coatings were not effective enough for the formation of a homogeneous and dense carbon layer of loose-fill thermal composite insulation materials and for stopping smoldering. Interesting results were observed in the loose-fill thermal composite insulation materials with the LG/EG and LG/TO/EG coatings (Figure 9g–j). In both compositions, the maximum temperature was achieved up to 60 min, i.e., 76 °C and 68 °C, respectively, but after 60 min, this temperature decreased to 32 °C and 35 °C, respectively, and no smoldering was observed in either of the two loose-fill thermal composite insulation materials. The obtained smoldering results of these multicomponent coatings confirm the previously presented flammability results, i.e., such coatings limit the spread of flames, fire, and the release of heat and ensure the formation of a suitable carbon layer, which limits the diffusion of oxygen from the air into the deeper layers of loose-fill thermal composite insulation materials.

**Figure 10:**
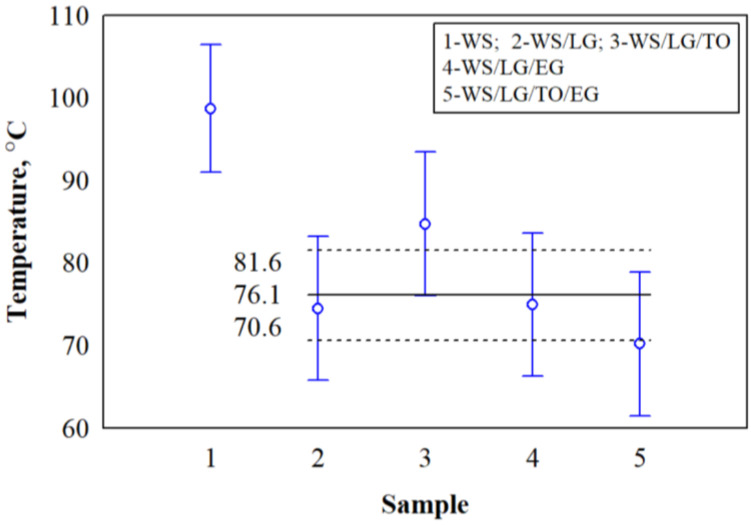
Statistical analysis of the obtained maximum temperatures during the smoldering test.

The analysis of the smoldering test, excluding uncoated WS, showed (Figure 10) that the average values of the maximum smoldering temperatures of WS/LG, WS/LG/TO, WS/LG/EG, and WS/LG/TO/EG did not differ. The obtained F criterion was 3.87, with *p* < 0.11. They indicate a statistically insignificant difference in the results of the subjects. Accordingly, the coefficient of determination was R^2^ = 0.744, and the corrected coefficient of determination was R^2^ = 0.552. Thus, the maximum temperature average value of the treated WS/LG, WS/LG/TO, WS/LG/EG, and WS/LG/TO/EG samples was ~76.1 ± 6.60 °C. Furthermore, the average value of the WS/LG/TO sample was greater than the upper confidence interval and reached ~84.8 ± 6.51 °C. Meanwhile, the average value of the WS/LG/TO/EG sample reached the lower confidence interval and was ~70.2 ± 3.46 °C.

## 4. Conclusions

The vacuum-based application of LG, LG/TO, LG/EG, and LG/TO/EG coatings for WS particles can enhance the thermal stability of loose-fill thermal composite insulation materials up to 190 °C at higher decomposition temperatures. The synergy between LG and EG is manifested by their ability to expand under the impact of temperature and form a double barrier at high temperatures.

The incorporation of TO into the LG/EG coating system assured the formation of a bubble-like film structure on the cracked LG surface and a dense char layer during combustion, which allowed the reduction of the pHRR value by a maximum of 80% and the TSR value by a maximum of 40%, suggesting that the LG/EG material protected TO from the adverse effects of the flame by changing its volume at high temperatures. Compared to the WS particles, the LG/TO and LG/TO/EG coatings increased CO_2_ emissions by two and four times, respectively, while the CO emissions of loose-fill thermal composite insulation materials changed only slightly due to both TO thermal instability and CO_2_ release during EG expansion.

Both LG and LG/TO coatings showed almost no change in their smoldering behavior because they are not sufficiently effective in forming a homogeneous and dense carbon layer alone. However, the synergistic effect between the three components in the LG/TO/EG coatings limited the spread of flames, fire, and the release of heat and ensured the formation of a suitable carbon layer, which confined the diffusion of oxygen from the air into the deeper layers of the loose-fill thermal composite insulation materials.

## Figures and Tables

**Table 1:** Compositions of loose-fill thermal insulation composites.

Sample	Component, wt.% by WS			
	WS	LG	TO	EG
WS	100	0	0	0
WS/LG	100	100	0	0
WS/LG/TO	100	100	10	0
WS/LG/EG	100	100	0	10
WS/LG/TO/EG	100	100	10	10

**Table 2:** Thermal stability results of loose-fill thermal insulation composite materials.

Composition	T_5%_, °C	T_50%_, °C	T_max._, °C				Char Residues at 900 °C, wt.%
			1	2	3	4	
WS	240	335	325	465	–	–	6.91
WS/LG	105	550	270	550	–	–	44.0
WS/LG/TO	175	505	275	570	465	–	33.4
WS/LG/EG	160	665	275	655	–	770	38.8
WS/LG/TO/EG	160	555	300	555	480	785	33.0

Note: T_5%_: temperature at 5% mass loss, °C; T_50%_: temperature at 50% mass loss, °C; T_max._: temperature at the respective endothermic peaks (Figure 1b), °C.

**Table 3:** Results from the cone calorimeter test of the loose-fill thermal composite insulation materials.

Composition	pHRR, kW/m^2^	TSR, m^2^/m^2^	COY, kg/kg	CO_2_Y, kg/kg
WS	98.6 ± 3.01	38.6 ± 4.95	0.35 ± 0.0404	1.01 ± 0.200
WS/LG	24.8 ± 1.97	43.5 ± 3.97	0.45 ± 0.0900	1.86 ± 0.201
WS/LG/TO	42.6 ± 3.00	61.2 ± 5.97	0.66 ± 0.0808	2.54 ± 0.299
WS/LG/EG	14.3 ± 2.00	23.3 ± 5.01	0.79 ± 0.0603	0.80 ± 0.110
WS/LG/TO/EG	18.0 ± 1.96	31.4 ± 2.95	0.38 ± 0.0400	4.30 ± 0.295
**Statistical Data**				
R^2^	0.996	0.917	0.910	0.978
Corrected R^2^	0.994	0.884	0.874	0.969
F	606	27.8	25.4	110
*p*	0	0	0	0

Note: F: Fisher’s distribution, *p*: significance level.

**Table 4:** Elemental composition of the char residues of loose-fill thermal composite insulation materials.

Composition	Elemental Composition, wt.%				
	C	O	Na	Si	S
WS	83.04	16.89	0	0.03	0.04
WS/LG	31.07	39.55	10.5	18.82	0.06
WS/LG/TO	54.71	34.96	6.63	3.67	0.03
WS/LG/EG	52.82	26.68	7.85	12.24	0.41
WS/LG/TO/EG	68.68	19.27	5.19	6.21	0.65

## Data Availability

The original contributions presented in the study are included in the article, further inquiries can be directed to the corresponding author.
